# An Overview of Systematic Reviews of Chinese Herbal Medicine for Parkinson's Disease

**DOI:** 10.3389/fphar.2019.00155

**Published:** 2019-03-05

**Authors:** Xin-Chun Jin, Li Zhang, Yong Wang, Hai-Bo Cai, Xian-Jun Bao, You-Yu Jin, Guo-Qing Zheng

**Affiliations:** ^1^Department of Neurology, The First People's Hospital of Wenling, The Affiliated Wenling Hospital of Wenzhou Medical University, Wenling, China; ^2^Department of Neurology, The Second Affiliated Hospital and Yuying Children's Hospital of Wenzhou Medical University, Wenzhou, China

**Keywords:** Chinese herbal medicine, traditional Chinese medicine, Parkinson's disease, overview, systematic review

## Abstract

Parkinson's disease (PD) is a high prevalence neurodegenerative disorder without a disease-modifying therapy. Up to now, a number of systematic reviews have been conducted to evaluate efficacy and safety of Chinese herbal Medicine (CHM) for PD patients. Here, we aimed to assess the methodological quality and reporting quality of systematic reviews using an overview, and then synthesize and evaluate the available evidence level of CHM for PD. Six databases were searched from inception to September 2018. The literatures were selected and data were extracted according to prespecified criteria. A Measurement Tool to Assess Systematic Reviews (AMSTAR) was used to evaluate the quality of methodology, and Grading of Recommendations Assessment, Development, and Evaluation (GRADE) to determine the evidence quality of the primary outcome measures. A total of 11 systematic reviews with 230 RCTs of CHM for PD were included. AMSTAR scores of the included reviews were range from 4 to 9. Compared with conventional western medicine (WCM), CHM paratherapy showed significant effect in improving UPDRS score, Webster scale score, PDQ-39, NMSQuest, CHM Syndrome Integral Scale, and PDSS. However, CHM monotherapy showed no difference relative to WCM according to various outcome measures. Adverse events were reported in 9 systematic reviews. The side effect in CHM paratherapy group was generally less than or lighter than that in WCM group. The quality of the evidence of primary outcomes was moderate (42%) to high (54%) according to the GRADE profiler. The present finding supported the use of CHM paratherapy for PD patients but we should treat the evidence cautiously because of the methodological flaws, whereas there is insufficient evidence of CHM monotherapy for PD.

## Introduction

Parkinson's disease (PD) is a common, chronic, and progressive neurodegenerative disorder resulting from the progressive loss of dopaminergic neurons in the substantia nigra and generates motor symptoms and non-motor symptoms (NMS) (Bohnen and Albin, 2011). Although the biochemical and molecular pathogenesis of the loss of dopaminergic neurons in PD has not been explicitly understood yet, it is thought to be involved in oxidative stress, mitochondrial dysfunction, and glutamate-mediated excitotoxicity and inflammation (Hirsch et al., 2013; Mullin and Schapira, 2015). Currently, there is no proven disease-modified cure for PD. Conventional medicine for PD, levodopa, is only symptomatic relief and always associated with levodopa-related motor fluctuation or dyskinesia. Thus, an increasing number of PD patients resort to complementary and alternative medicine (CAM), estimating the prevalence of CAM use for PD to be between 25.7 and 76% according to the epidemiological data from seven separate countries (Wang et al., 2013; Pan et al., 2018).

Traditional Chinese medicine (TCM), one of main forms of CAM has played an indispensable role in medical care of PD patients for thousands of years in China, and currently is extended to use worldwide (Zheng, 2009; Wang et al., 2011, 2013). Chinese herbal medicine (CHM) is main pharmacological therapy of TCM. The herbal extracts and their biocompounds exert antioxidant, anti-apoptotic, and anti-inflammatory effects, which contribute to avoiding neuronal loss, acting on the biosynthesis of dopamine and its metabolites, and preventing D2 receptors' hypersensitivity (da Costa et al., 2017). In the past years, a number of systematic reviews have been conducted to evaluate the potential therapeutic benefits of CHM for PD (Chung et al., 2006; Kim et al., 2012; Wang et al., 2012; Huo and Yu, 2014; Wen et al., 2014; Zhang et al., 2014, 2015; Cui and Liu, 2015; Zhang, 2015; Wei et al., 2017; Shan et al., 2018), but their conclusions are inconsistent because of the quality of primary studies or methodological flaws. In addition, an overview of systematic reviews (SRs) is a novel tool to address a specific, focused question, relevant to policy or practice, and synthesize evidence from multiple SRs into a single, useful file that can be used to guide health care professionals and policy makers (Thomson et al., 2010; Baker et al., 2014). Thus, we conducted an overview to critically assess the methodological quality and reporting quality of SRs, and then, to synthesize and evaluate the available evidence level of CHM for PD.

## Methods

### Search Strategy

Electronic literature was searched in the following databases from inception to September 31, 2018 without language restrictions: Pubmed, EMBASE, Web of Sciences, China National Knowledge Infrastructure, VIP Journals Database, and Wan fang Med Online Database. The keywords used were as follows: “Traditional Chinese Medicine OR herbal medicine” AND “systematic review OR meta-analysis” AND “Parkinson's Disease” (Parkinson's Disease as a mesh term). For Chinese database, above search terms were used in Chinese accordingly. The following search strategy was used for PubMed and was modified to suit other databases.

#1. Parkinson's Disease [mh]

#2. traditional Chinese medicine [tiab]

#3. herbal medicine [tiab]

#4. systematic review [tiab]

#5. meta-analysis [tiab]

#6. #2 OR #3

#7. #4 OR #5

#8. #1 AND #6 AND #7

### Eligibility Criteria

Type of study: We included SRs of CHM for PD that met the following criteria: (1) evaluated the effects of CHM on PD compared with western conventional medicine (WCM); (2) provided a clearly definition of clinical question, eligibility criteria, and searching strategies; (3) reported at least one results of desired outcome. SRs with insufficient information for methods section, quality evaluation and methodology study were excluded.

Type of participants: Participants were of any age or sex with a confirmed diagnosis of PD based on at least one of following criteria: (1) the UK Brain Bank criteria (Hughes et al., 1992); (2) Chinese National Diagnosis Standard (CNDS) for PD in 1984 (Wang, 1985); (3) CNDS updated version in 2006 for PD (Zhang, 2006); (4) other formal comparable criteria.

Type of intervention: CHM or CHM paratherapy were used in the treatment groups, regardless of the form of the drug, dosage, frequency or duration of the treatment. Comparator interventions were placebo or WCM.

Type of outcome measures: The primary outcomes were total Unified Parkinson's Disease Rating Scale (UPDRS) score, Webster scale, Parkinson's Disease Questionnaire-39 (PDQ-39), and Non-motor Symptoms Questionnaire (NMSQuest). The UPDRS was the major rating scale assessing severity of symptoms of PD. The UPDRS scale consists of the following four segments: Part I (mentation, behavior, and mood) addresses mental dysfunction and mood; Part II (activities of daily living, ADL) assesses motor disability; Part III (motor section) evaluates motor impairment; Part IV (complications) assesses treatment related motor and non-motor complications. The secondary outcomes were Parkinson's Disease Sleep Scale (PDSS), Hamilton depression rating scale (HAM-D), CHM syndrome integral scale, the 36-Item Short Form Health Survey (SF-36), and adverse reactions.

### Study Selection and Data Collection

Two investigators (XC-J and LZ) independently screened the title and abstract to select potential references. Full articles were obtained for potentially useful studies. The two investigators independently read the whole articles and made a final decision. The data collection from the studies included author name, year of publication, country of first author, number of primary studies and samples, overall conclusion, meta-analysis, outcome measures. Disagreement between two researchers was resolved by discussion with the third author. If the critical data were missing or only expressed graphically, we tried to contact authors for further information or calculated by ourselves if available.

### Assessing the Quality of SRs

A Measurement Tool to Assess SRs (AMSTAR) (Shea et al., 2007), which consists of 11 items was used to evaluate the methodological quality of all included SRs. For each item, a judgement of “Yes,” “No,” “Can't answer” or “Not applicable” was assigned according to judgment criteria of AMSTAR. The number of “yes” will be counted as the total score of AMSTAR. A total score of 4 or less was considered as indication of low quality, a total score of 5 to8 means moderate quality and a total score of 9 or more suggests high quality (Monasta et al., 2010; Jaspers et al., 2011). Each SR was assessed by two researchers (XC-J and LZ) independently, and any disagreements were resolved by discussing with a third author (GQZ).

### Assessing the Quality of Evidence

For the primary outcome measures with detailed information, GRADE (Guyatt et al., 2008) was used to evaluate the quality of evidence following the GRADE handbook (Guyatt et al., 2008) by two researchers (XC-J and LZ) independently and disagreements were resolved by a third author (GQZ). GRADE classified the quality of evidence into four levels: high, moderate, low, and very low. We judged evidence as high quality when we were highly confident that the true effect lay close to that of the estimate of the effect; we judged evidence as moderate quality when we considered that the true effect was likely to be close to the estimate of the effect, but there was a possibility that it was substantially different; we judged evidence to be low or very low quality when the true effect might be substantially different from the estimate of the effect.

### Data Synthesis

A narrative description of the included SRs was conducted. Review-level summaries for all the primary and secondary outcomes from the included SRs were tabulated. We extracted and reported pooled effect sizes, when outcomes were meta-analyzed within a SR. If there was no quantitative pooling of effect sizes, we reported results with a standardized language indicating direction of effect and statistical significance. Risk ratio (RR) with 95% confidence interval (CI) was involved when summary the dichotomous outcomes, while weighted mean difference (WMD) or standard mean difference (SMD) and 95% CI was involved when summary the continuous data. The heterogeneity of each included SR was also summary and analyzed, which was detected by *I*^2^ and Chi^2^ tests.

## Results

### Description of the Screening Process

**Figure 1:**
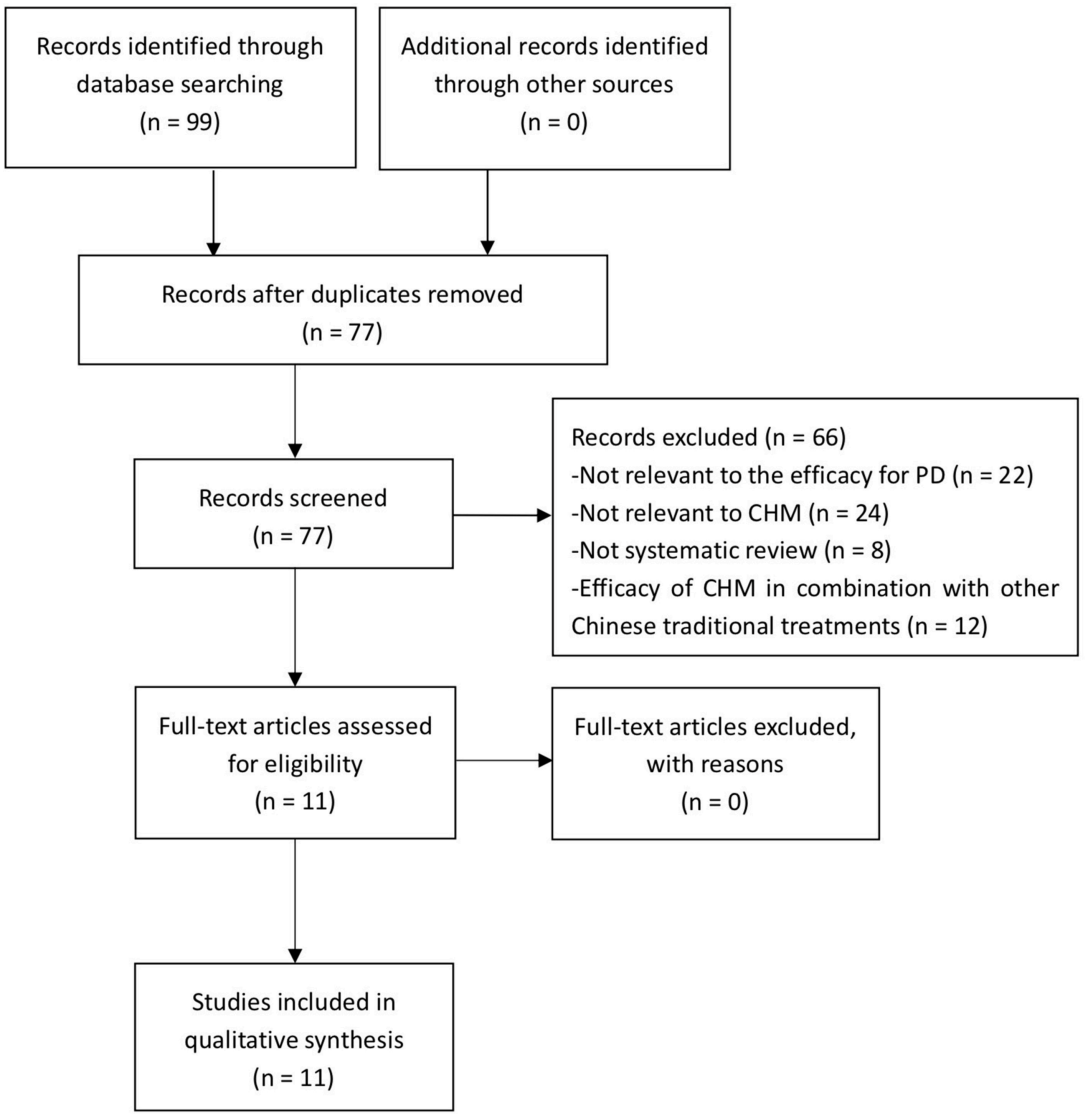
PRISMA 2009 flow diagram.

A total of 99 studies were retrieved, and of which 22 studies were excluded because of duplicates. After screening titles and abstracts, 66 studies were excluded because they are not relevant to the efficacy for PD, or not relevant to CHM, or not SR, or in combination with other TCM therapeutic modalities. Ultimately, 11 eligible studies were included in the present study. The process of screening is presented in a flow diagram (Figure 1).

### Study Characteristics

Eleven SRs with 230 randomized controlled trials (RCTs) were included in the present study. Ten SRs were published journal articles, while only one SR was academic dissertation (Zhang, 2015). Four SRs were published in Chinese (Huo and Yu, 2014; Wen et al., 2014; Cui and Liu, 2015; Zhang, 2015) and 7 others in English from 2006 to 2018, in which 8 SRs published in recent 5 years. The first authors of 10 studies were from China and affiliated to academic institutions, while the first author of only one study (Kim et al., 2012) was from Korea. All included SRs evaluated the efficacy of CHM for PD. Two studies (Kim et al., 2012; Shan et al., 2018) compared CHM with placebo. Four studies (Chung et al., 2006; Kim et al., 2012; Huo and Yu, 2014; Wen et al., 2014) compared CHM therapy with WCM. Comparing CHM paratherapy with WCM were conducted in 10 studies (Chung et al., 2006; Kim et al., 2012; Wang et al., 2012; Wen et al., 2014; Zhang et al., 2014, 2015; Cui and Liu, 2015; Zhang, 2015; Wei et al., 2017; Shan et al., 2018). The number of RCTs included in SRs ranged from 9 to 64. The overall quality of primary studies was poor according to the Jadad score (Huo and Yu, 2014; Wen et al., 2014; Cui and Liu, 2015; Zhang, 2015) or Cochrane risk of bias tool (Chung et al., 2006; Kim et al., 2012; Wang et al., 2012; Zhang et al., 2014, 2015; Wei et al., 2017; Shan et al., 2018). Nine SRs conducted meta-analysis, while the other 2 (Chung et al., 2006; Kim et al., 2012) did not. The characteristics of the included SRs were summarized in Table 1.

**Table 1:** Study characteristics of included systematic reviews.

**References**	**No. of primary studies**	**Quality of primary studies**	**Overall conclusion**	**Meta-analysis**	**Outcome measures**	**Quality of review (AMSTAR)**
Cui and Liu (2015)	19(1352)	Poor	…is better than…	CHM+CWM vs. CWM: the total score of UPDRS [SMD = −7.32, 95%CI(−8.50, −6.13), *P* < 0.00001; heterogeneity: Chi^2^ = 3.74, *P* = 0.81, *I*^2^ = 0%], UPDRS I [WMD = −0.76, 95%CI(−1.30, −0.23), *P* = 0.005; heterogeneity: Chi^2^ = 16.46, *P* = 0.002, *I*^2^ = 76%], UPDRS II [WMD = −3.07, 95%CI(−4.18, −1.97), *P* < 0.00001; heterogeneity: Chi^2^ = 6.06, *P* = 0.19, *I*^2^ = 34%], UPDRS III [WMD = −4.09, 95%CI(−5.05, −3.12), *P* < 0.00001; heterogeneity: Chi^2^ = 2.52, *P* = 0.93, *I*^2^ = 0%], UPDRS II and UPFRS III [WMD = −7.01, 95%CI(−11.61, −2.41), *P* = 0.003; heterogeneity: Chi^2^ = 7.36, *P* = 0.06, *I*^2^ = 59%], UPDRS IV [SMD = −0.63, 95%CI(−1.00, −0.26), *P* = 0.0009; heterogeneity: Chi^2^ = 27.05, *P* = 0.0001, *I*^2^ = 78%]	UPDRS	7
Huo and Yu (2014)	22(1704)	Poor	…can relieve… …maybe superior to…	CHM+CWM vs. CWM: the total score of UPDRS [WMD = −1.59, 95%CI(−2.59, −0.59), *P* = 0.002; heterogeneity: Chi2 = 2.82, *P* = 0.42, *I*^2^ = 0%], UPDRS II [WMD = −1.08, 95%CI(−1.53, −0.63), *P* < 0.00001; heterogeneity: Chi^2^ = 5.17, *P* = 0.40, *I*^2^ = 3.3%], UPDRS III [WMD = −1.15, 95%CI(−1.69, −0.61), *P* < 0.0001; heterogeneity: Chi^2^ = 6.04, *P* = 0.30, *I*^2^ = 17.2%]	UPDRS Adverse events	6
Wen et al. (2014)	17(1461)	Poor	…is safe and effective… …maybe superior to…	CHM vs. CWM: the total score of UPDRS [WMD = 1.41, 95%CI(−0.66, 3.47), *P* > 0.05; heterogeneity: Chi^2^ = 0.26, df = 1, *I*^2^ = 0%] CHM+CWM vs. CWM: the total score of UPDRS [WMD = 5.95, 95%CI(4.37, 7.42), *P* < 0.01; heterogeneity: Chi^2^ = 2.16, df = 4, *I*^2^ = 0%], UPDRS II [WMD = 2.13, 95%CI(1.62, 2.64), *P* < 0.01; heterogeneity: Chi^2^ = 4.94, df = 5, *I*^2^ = 30.7%], UPDRS III [WMD = 1.95, 95%CI(0.81, 2.42), *P* < 0.01; heterogeneity: Chi^2^ = 3.12, df = 5, *I*^2^ = 0%], UPDRS IV [SMD = 0.64, 95%CI(0.32, 0.97), *P* < 0.01; heterogeneity: Chi^2^ = 5.37, df = 4, *I*^2^ = 32.8%]	UPDRS UPDRS Webster scale Adverse events	5
Zhang (2015)	18(1504)	Poor	…is safe and effective… …maybe superior to…	CHM+CWM vs. CWM: the total score of UPDRS [WMD = −4.67, 95%CI(−5.94, −3.40), *P* < 0.0001; heterogeneity: Chi^2^ = 13.29, *P* = 0.15, *I*^2^ = 32%], UPDRS II [WMD = −1.16, 95%CI(−1.35, −0.98), *P* < 0.00001; heterogeneity: Chi^2^ = 7.16, *P* = 0.52, *I*^2^ = 0%], UPDRS III [WMD = −3.05, 95%CI(−3.48, −2.63), *P* < 0.00001; heterogeneity: Chi^2^ = 3.26, *P* = 0.86, *I*^2^ = 0%], CHM syndrome integral scale [WMD = −3.04, 95%CI(−3.83, −2.25), *P* < 0.00001; heterogeneity: Chi^2^ = 2.83, *P* = 0.24, *I*^2^ = 29%]	UPDRS Webster scale Adverse events CHM syndrome integral scale	8
Zhang et al. (2014)	10(582)	Poor	…may be beneficial to…	CHM+CWM vs. CWM: the total score of UPDRS [WMD = −7.35, 95%CI(−11.24, −3.47), *P* = 0.000; heterogeneity: *P* = 0.034, *I*^2^ = 65.5%]	UPDRS Adverse events	8
Wang et al. (2012)	19(1371)	Poor	…may potentially alleviate…	CHM+CWM vs. CWM: the total score of UPDRS [WMD = −6.09, 95%CI(−8.08, −4.10), *P* < 0.001; heterogeneity: Chi^2^ = 4.25, *P* = 0.89, *I*^2^ = 0%], UPDRS I [WMD = −0.33, 95%CI(−0.58, −0.08), *P* < 0.001; heterogeneity: Chi^2^ = 3.69, *P* = 0.45, *I*^2^ = 0%], UPDRS II [WMD = −2.18, 95%CI(−3.03, −1.33), *P* < 0.001; heterogeneity: Chi^2^ = 3.26, *P* = 0.92, *I*^2^ = 0%], UPDRS III [WMD = −2.35, 95%CI(−4.61, −0.08), *P* < 0.05; heterogeneity: Chi^2^ = 89.22, *P* < 0.00001, *I*^2^ = 88%], UPDRS IV [WMD = −0.51, 95%CI(−0.83, −0.20), *P* < 0.05; heterogeneity: Chi^2^ = 5.21, *P* = 0.52, *I*^2^ = 0%]	UPDRS Adverse events	7
Chung et al. (2006)	9(503)	Poor	…is insufficient to…	CHM+CWM vs. CWM: without meta-analysis CHM vs. CWM: without meta-analysis	UPDRS Adverse events UPDRS PDQ-39 Webster scale Adverse events	4
Kim et al. (2012)	64(4024)	Poor	…is no conclusive evidence…	CHM vs. placebo: without meta-analysis CHM vs. CWM: without meta-analysis CHM+CWM vs. CWM: without meta-analysis	UPDRS UPDRS Webster scale UPDRS NMSQuest	7
Zhang et al. (2015)	27(2314)	Poor	…potential superiority of…	CHM+CWM vs. CWM: the total score of UPDRS [WMD = 6.18, 95%CI(5.06, 7.31), *P* < 0.00001; heterogeneity: Chi^2^ = 8.93, *P* = 0.54, *I*^2^ = 0%], UPDRS I [SMD = 0.68, 95%CI(0.38, 0.98), *P* < 0.00001; heterogeneity: Chi^2^ = 21.85, *P* = 0.009, *I*^2^ = 59%], UPDRS II [WMD = 2.41, 95%CI (1.66, 2.62), *P* < 0.00001; heterogeneity: Chi^2^ = 10.98, *P* = 0.61, *I*^2^ = 0%], UPDRS III [WMD = 2.45, 95%CI(2.03, 2.86), *P* < 0.00001; heterogeneity: Chi^2^ = 25.23, *P* = 0.07, *I*^2^ = 37%], UPDRS IV [WMD = 0.32, 95%CI(0.15, 049), *P* = 0.0002; heterogeneity: Chi^2^ = 9.78, *P* = 0.46, *I*^2^ = 0%]	UPDRS Adverse events	9
Wei et al. (2017)	11(869)	Poor	…has potential therapeutic benefits…	CHM+CWM vs. CWM: the total score of UPDRS [SMD = −0.36, 95%CI (−0.53, −0.20), *P* < 0.05; heterogeneity: Chi^2^ = 2.62, *P* = 0.85, *I*^2^ = 0%], UPDRS I [SMD = −0.40, 95%CI(−0.71, −0.09), *P* = 0.01; heterogeneity: Chi^2^ = 0.05, *P* = 0.82, *I*^2^ = 0%], UPDRS II [SMD = −0.47, 95%CI (−0.69, −0.25), *P* < 0.01; heterogeneity: Chi^2^ = 1.36, *P* = 0.85, *I*^2^ = 0%], UPDRS III [SMD = −0.35, 95%CI(−0.57, −0.13), *P* = 0.002; heterogeneity: Chi^2^ = 1.51, *P* = 0.83, *I*^2^ = 0%], UPDRS IV [SMD = −0.32, 95%CI(−0.60, −0.03), *P* = 0.03; heterogeneity: Chi^2^ = 3.43, *P* = 0.18, *I*^2^ = 42%]; PDQ-39 [SMD = −0.35, 95%CI(−0.59, −0.12), *P* < 0.05; heterogeneity: Chi^2^ = 0.12, *P* = 0.99, *I*^2^ = 0%]; CHM syndrome integral scale [SMD = −0.73, 95%CI(−1.05, −0.41), *P* < 0.05; heterogeneity: Chi^2^ = 0.98, *P* = 0.32, *I*^2^ = 0%]	UPDRS PDQ-39 CHM syndrome integral scale Adverse events	9
Shan et al. (2018)	14(1311)	Poor	…supported the complementary use of…	CHM+CWM vs. CWM: the total score of UPDRS [WMD = −5.43, 95%CI(−8.01, −2.86), *P* < 0.0001; heterogeneity: Chi^2^ = 2.59, *P* = 0.76, *I*^2^ = 0%], UPDRS I [WMD = −0.30, 95%CI(−0.54, −0.06), *P* = 0.02; heterogeneity: Chi^2^ = 3.21, *P* = 0.52, *I*^2^ = 0%], UPDRS II [WMD = −2.21, 95%CI(−3.19, −1.22), *P* < 0.0001; heterogeneity: Chi^2^ = 3.46, *P* = 0.84, *I*^2^ = 0%], UPDRS III [WMD = −3.26, 95%CI(−4.36, −2.16), *P* < 0.00001; heterogeneity: Chi^2^ = 1.88, *P* = 0.98, *I*^2^ = 0%], UPDRS IV [WMD = −0.18, 95%CI(−0.37, −0.01), *P* = 0.06; heterogeneity: Chi^2^ = 5.76, *P* = 0.33, *I*^2^ = 13%]; PDQ-39 [WMD = −7.65, 95%CI(−11.46, −3.83), *p* < 0.0001; heterogeneity: Chi^2^ = 0.12, *P* = 0.94, *I*^2^ = 0%]; NMSQuest [WMD = −9.19, 95%CI(−13.11, −5.28), *P* < 0.00001; heterogeneity: Chi^2^ = 0.56, *P* = 0.45, *I*^2^ = 0%]; PDSS [WMD = 10.69, 95%CI(8.86, 12.53), *P* < 0.00001; heterogeneity: Chi^2^ = 0.48, *P* = 0.49, *I*^2^ = 0%] CHM vs. placebo: total UPDRS score (*P* > 0.05), UPDRS II (*P* > 0.05), UPDRS III (*P* > 0.05)	UPDRS PDQ-39 NMSQuest PDSS Adverse events UPDRS	9

*CHM, Chinese herbal medicine; CWM, conventional western medicine; UPDRS, The Unified Parkinson's disease Rating Scale; PDQ-39, Parkinson's Disease Questionnaire-39; NMSQuest, Non-Motor Symptoms Questionnaire; PDSS, Parkinson's Disease Sleep Scale; SF36, the 36-Item Short Form Health Survey; HAM-D, The Hamilton Depression Rating Scale*.

### Description of the CHM Formulas and High-Frequency Used Herbs

Eight out of the 11 SRs summarized the CHM formulas and reported a wide range of CHM formulas. A total of 52 CHM formulas were used in these studies. The top 3 most frequently used formulas were BushenHuoxue granule, Guiling Pa'an granule, Xifeng Dingchan granule. The top 10 high-frequency used herbs for PD in included studies were *Rhizoma Ligustici Chuanxiong, Radix Paeoniae Alba, Rhizoma Acori Tatarinowii, Radix Angelicae Sinensis, Fructus Corni, Radix Polygoni Multiflori, Radix Changii, Rhizoma Coptidis, Rhizoma Gastrodiae, Radix Glycyrrhizae*. The details of these 10 herbs were generalized in Table 2.

**Table 2:** Details of high-frequency used herbs for PD.

**Chinese name**	**Pharmaceutical name**	**Species**	**Family**	**Record**
Chuanxiong	*Rhizoma Ligustici Chuanxiong*	*Ligusticum striatum* DC.	*Apiaceae*	–
Baishao	*Radix Paeoniae Alba*	*Paeonia lactiflora* Pall.	*Paeoniaceae*	–
Shichangpu	*Rhizoma Acori Tatarinowii*	*Acorus tatarinowii* Schott	*Acoraceae*	2,337
Danggui	*Radix Angelicae Sinensis*	*Angelica sinensis* (Oliv.) Diels	*Apiaceae*	–
Shanzhuyu	*Fructus Corni*	*Cornus officinalis* Siebold & Zucc.	*Cornaceae*	47,459
Heshouwu	*Radix Polygoni Multiflori*	*Polygonum multiflorum* Thunb.	*Polygonaceae*	–
Dangshen	*Radix Changii*	*Changium smyrnioides* H. Wolff	*Apiaceae*	–
Huanglian	*Rhizoma Coptidis*	*Coptis chinensis* Franch.	*Ranunculaceae*	–
Tianma	*Rhizoma Gastrodiae*	*Gastrodia elata* Blume	*Orchidaceae*	88,817
Zhigancao	*Radix Glycyrrhizae*	*Glycyrrhiza uralensis* Fisch.	*Eguminosae*	32,406

### Assessing the Quality of SRs

AMSTAR scale was used to evaluate the methodological quality of the included SRs. All of the included SRs were not registered in advance and did not provide a list of included and excluded studies. One SR (Huo and Yu, 2014) did not perform a comprehensive literature search, 2 SRs (Huo and Yu, 2014; Wen et al., 2014) did not search gray literature, 3 studies (Chung et al., 2006; Kim et al., 2012; Wang et al., 2012) did not assess and document the scientific quality of the included studies, and the scientific quality of the included studies did not used appropriately in formulating conclusions in them. Two SRs (Chung et al., 2006; Wen et al., 2014) did not appropriately explain the findings of studies, 3 SRs (Chung et al., 2006; Wen et al., 2014; Cui and Liu, 2015) did not assess the likelihood of publication bias, and 6 SRs (Chung et al., 2006; Huo and Yu, 2014; Wen et al., 2014; Zhang et al., 2014; Cui and Liu, 2015; Zhang, 2015) did not state the conflicts of interest. For overall scores, 3 SRs achieved high quality with scoring 9 points of AMSTAR (Zhang et al., 2015; Wei et al., 2017; Shan et al., 2018); one was low quality with scoring 4 points (Chung et al., 2006); the quality of the remaining 7 studies were moderate. Among them, 3 SRs scored 7 points (Kim et al., 2012; Wang et al., 2012; Cui and Liu, 2015), 2 scored 8 points (Zhang et al., 2014; Zhang, 2015), 1 scored 5 points (Wen et al., 2014), and 1 scored 6 points (Huo and Yu, 2014). The details of the assessment of the quality of SRs are listed in Table 3.

**Table 3:** A measurement tool to assess systematic reviews (AMSTAR) for the included systematic reviews.

**References**	**1**	**2**	**3**	**4**	**5**	**6**	**7**	**8**	**9**	**10**	**11**	**12**
Cui and Liu (2015)	–	+	+	+	–	+	+	+	+	–	–	7
Huo and Yu (2014)	–	+	–	–	–	+	+	+	+	+	–	6
Wen et al. (2014)	–	+	+	–	–	+	+	+	–	–	–	5
Zhang (2015)	–	+	+	+	–	+	+	+	+	+	–	8
Wang et al. (2012)	–	+	+	+	–	+	–	–	+	+	+	7
Chung et al. (2006)	–	+	+	+	–	+	–	–	–	–	–	4
Kim et al. (2012)	–	+	+	+	–	+	–	–	+	+	+	7
Zhang et al. (2014)	–	+	+	+	–	+	+	+	+	+	–	8
Zhang et al. (2015)	–	+	+	+	–	+	+	+	+	+	+	9
Wei et al. (2017)	–	+	+	+	–	+	+	+	+	+	+	9
Shan et al. (2018)	–	+	+	+	–	+	+	+	+	+	+	9

*1. Was an “a priori” design provided? 2. Was there duplicate study selection and data extraction? 3. Was a comprehensive literature search performed? 4. Was the status of publication (i.e., gray literature) used as an inclusion criterion? 5. Was a list of studies (included and excluded) provided? 6. Were the characteristics of the included studies provided? 7. Was the scientific quality of the included studies assessed and documented? 8. Was the scientific quality of the included studies used appropriately in formulating conclusions? 9. Were the methods used to combine the findings of studies appropriate? 10. Was the likelihood of publication bias assessed? 11. Was the conflict of interest included? 12. Overall scores. – refers to 0 point, + refers to 1 point, N/A refers to not available*.

### Effectiveness

#### UPDRS I

##### CHM paratherapy vs. WCM

Five SRs (Wang et al., 2012; Cui and Liu, 2015; Zhang et al., 2015; Wei et al., 2017; Shan et al., 2018) assessed the UPDRS I score and all of them indicated that CHM combined with WCM is better than that of WCM (*P* < 0.05). Meta-analysis was conducted in all of 5 SRs. The heterogeneity of 3 SRs (Wang et al., 2012; Wei et al., 2017; Shan et al., 2018) was acceptable with *I*^2^ < 50%, while in 2 SRs (Cui and Liu, 2015; Zhang et al., 2015) was high with *I*^2^ > 50%. The reason of high heterogeneity was not explained in both of the 2 SRs. The details of WMD or SMD, 95% CI, and heterogeneity were generalized in Table 1.

#### UPDRS II

##### CHM vs. placebo

One SR (Shan et al., 2018) showed that the efficacy of CHM monotherapy was similar to placebo according to UPDRS II (*P* > 0.05).

##### CHM paratherapy vs. WCM

UPDRS II was assessed in 9 SRs (Chung et al., 2006; Wang et al., 2012; Huo and Yu, 2014; Wen et al., 2014; Cui and Liu, 2015; Zhang, 2015; Zhang et al., 2015; Wei et al., 2017; Shan et al., 2018). All of them indicated CHM paratherapy significantly improved UPDRS II compared with WCM (*P* < 0.05). Eight (Wang et al., 2012; Huo and Yu, 2014; Wen et al., 2014; Cui and Liu, 2015; Zhang, 2015; Zhang et al., 2015; Wei et al., 2017; Shan et al., 2018) out of 8 SRs conducted meta-analysis, and the heterogeneity of each one was low with I^2^ < 50%.

#### UPDRS III

##### CHM vs. placebo

One SR (Shan et al., 2018) showed that the efficacy of CHM monotherapy was similar to placebo according to UPDRS III (*P* > 0.05).

##### CHM paratherapy vs. WCM

UPDRS III was assessed in 9 SRs (Chung et al., 2006; Wang et al., 2012; Huo and Yu, 2014; Wen et al., 2014; Cui and Liu, 2015; Zhang, 2015; Zhang et al., 2015; Wei et al., 2017; Shan et al., 2018). All of them indicated CHM paratherapy significantly improved UPDRS III compared with WCM (*P* < 0.05). Eight (Wang et al., 2012; Huo and Yu, 2014; Wen et al., 2014; Cui and Liu, 2015; Zhang, 2015; Zhang et al., 2015; Wei et al., 2017; Shan et al., 2018) out of 9 SRs conducted meta-analysis. The heterogeneity of 6 SRs (Huo and Yu, 2014; Cui and Liu, 2015; Zhang, 2015; Zhang et al., 2015; Wei et al., 2017; Shan et al., 2018) was acceptable with I^2^ < 50%, while in 2 SRs (Wang et al., 2012; Wen et al., 2014) was high with I^2^ > 50%. In (Wen et al., 2014), the high heterogeneity related to the different participants included in one trail. After removing the trail, the UPDRS III appeared homogeneous (WMD = 1.95, 95%CI(0.81, 2.42), *P* < 0.01; heterogeneity: Chi^2^ = 3.12, df = 5, *I*^2^ = 0%). The reason of high heterogeneity was not explained in Zhang et al. (2015)'s study.

#### UPDRS IV

##### CHM paratherapy vs. WCM

UPDRS IV was assessed in 6 SRs (Wang et al., 2012; Wen et al., 2014; Cui and Liu, 2015; Zhang et al., 2015; Wei et al., 2017; Shan et al., 2018). Five SRs (Wang et al., 2012; Wen et al., 2014; Cui and Liu, 2015; Zhang et al., 2015; Wei et al., 2017) indicated that CHM paratherapy significantly improved UPDRS IV compared with WCM (*P* < 0.05). One SR showed no difference between CHM paratherapy and WCM for improving UPDRS IV (WMD = −0.18, 95%CI (−0.37, −0.01), *P* = 0.06; heterogeneity: Chi^2^ = 5.76, *P* = 0.33, *I*^2^ = 13%). The heterogeneity of 5 SRs (Wang et al., 2012; Wen et al., 2014; Zhang et al., 2015; Wei et al., 2017; Shan et al., 2018) was acceptable with *I*^2^ < 50%, while in 1 SR (Cui and Liu, 2015) was high (SMD = −0.63, 95%CI(−1.00, −0.26), *P* = 0.0009; heterogeneity: Chi^2^ = 27.05, *P* = 0.0001, *I*^2^ = 78%). However, Cui and Liu (2015) did not explain the high heterogeneity.

#### Total Score of UPDRS

##### CHM vs. placebo

In one SR (Kim et al., 2012), CHM showed significant improvement in total UPDRS score after treatment (*P* < 0.05). One SR (Shan et al., 2018) showed that the efficacy of CHM monotherapy was similar to placebo according to total UPDRS score (*P* > 0.05).

##### CHM vs. WCM

Total UPDRS score was assessed in 3 SRs (Chung et al., 2006; Kim et al., 2012; Wen et al., 2014). One SRs (Kim et al., 2012) indicated CHM monotherapy significantly improved total UPDRS score compared with WCM (*P* < 0.05), while two SRs (Chung et al., 2006; Wen et al., 2014) showed that the efficacy of CHM monotherapy was similar to WCM (*P* > 0.05). Meta-analysis was conducted in 1 (Wen et al., 2014) out of 3 SRs with no heterogeneity (*I*^2^ = 0). See Table 1 for more information.

##### CHM paratherapy vs. WCM

Total UPDRS score was assessed in 11 SRs (Chung et al., 2006; Kim et al., 2012; Wang et al., 2012; Huo and Yu, 2014; Wen et al., 2014; Zhang et al., 2014, 2015; Cui and Liu, 2015; Zhang, 2015; Wei et al., 2017; Shan et al., 2018). All 11 SRs showed CHM paratherapy was better than that of WCM according to total UPDRS score (*P* < 0.05). Nine SRs (Wang et al., 2012; Huo and Yu, 2014; Zhang et al., 2014, 2015; Cui and Liu, 2015; Zhang, 2015; Wei et al., 2017; Shan et al., 2018) conducted meta-analysis; among which 7 SRs was considered to have low heterogeneity (I^2^ < 50%), while 2 SRs (Wen et al., 2014; Zhang et al., 2014) had high heterogeneity (I^2^ > 50%). In Wen et al.'s SR (2014), the high heterogeneity related to the different participants included in one trail. After removing the trail, the UPDRS III appeared homogeneous (WMD = 5.95, 95%CI (4.37, 7.42), *P* < 0.01; heterogeneity: Chi^2^ = 2.16, df = 4, *I*^2^ = 0%). The reason of high heterogeneity was not explained in Zhang et al. (2014)'s study.

#### Webster Scale

##### CHM vs. WCM

Webster scale score was assessed in 2 SRs (Chung et al., 2006; Kim et al., 2012). In (Chung et al., 2006)'s SR (2006), two trails reported the improvement in the overall Webster scale scoring. However, flaws in design and statistical analysis in these two studies limited the reliability of their conclusions. In (Kim et al., 2012)'s SR (2012), three CHM formulas showed significant effect for improving Webster score.

##### CHM paratherapy vs. WCM

One SR (Chung et al., 2006) showed the significant effect of CHM paratherapy for improving Webster score compared with WCM. Three out of 4 trails included in (Kim et al., 2012)' SR (2012) indicated that combination therapy is better than that of WCM.

#### PDQ-39

##### CHM vs. WCM

One SR (Kim et al., 2012) indicated that the efficacy of CHM monotherapy was similar to WCM according to PDQ-39 (*P* > 0.05).

##### CHM paratherapy vs. WCM

Two SRs (Wei et al., 2017; Shan et al., 2018) assessed PDQ-39 and conducted meta-analysis. Both 2 SRs indicated a significant effect of CHM paratherapy for improving PDQ-39 compared with WCM (*P* < 0.05). The heterogeneity of Wei et al.'s study was low (SMD = −0.35, 95% CI (−0.59, −0.12), *P* < 0.05; heterogeneity: Chi^2^ = 0.12, *P* = 0.99, *I*^2^ = 0%), while was high in Shan et al.'s study (*I*^2^ >84%). After removing imbalanced baseline, the outcome measures appeared homogeneous (WMD = −7.65, 95%CI (−11.46, −3.83), *P* < 0.0001; heterogeneity: Chi^2^ = 0.12, *P* = 0.94, *I*^2^ = 0%).

#### NMSQuest

##### CHM paratherapy vs. WCM

One SR (Shan et al., 2018) indicated that CHM paratherapy was significant better effects according to NMSQuest (WMD = −9.19, 95% CI (−13.11, −5.28), *P* < 0.00001; heterogeneity: Chi2 = 0.56, P = 0.45, *I*^2^ = 0%). In contrary, one SR showed there was no significant difference comparing CHM paratherapy with WCM in NMSQuest value (*P* > 0.05).

#### CHM Syndrome Integral Scale, PDSS

##### CHM paratherapy vs. WCM

CHM Syndrome Integral Scale was assessed in 2 SRs (Zhang, 2015; Wei et al., 2017). Meta-analysis of these 2 SRs indicated a significant effect of CHM paratherapy for improving CHM Syndrome compared with WCM (Zhang, 2015) WMD = −3.04, 95%CI(−3.83, −2.25), *P* < 0.00001; heterogeneity: Chi^2^ = 2.83, *P* = 0.24, *I*^2^ = 29%; (Wei et al., 2017): SMD = −0.73, 95%CI(−1.05, −0.41), *P* < 0.05; heterogeneity: Chi^2^ = 0.98, *P* = 0.32, *I*^2^ = 0%).

PDSS was assessed in 1 SR (Shan et al., 2018). Meta-analysis showed that CHM paratherapy was better than that of WCM according to PDSS (WMD = 10.69, 95% CI (8.86, 12.53), *P* < 0.00001; heterogeneity: Chi^2^ = 0.48, P = 0.49, *I*^2^ = 0%).

#### Adverse Events

One SR (Chung et al., 2006) evaluated adverse events associated with CHM, including dry mouth, altered taste, musculoskeletal pain, diarrhea/loose stool, constipation, and dizziness. These adverse events were more common in the WCM group than that in the CHM group.

Nine SRs (Chung et al., 2006; Wang et al., 2012; Wen et al., 2014; Zhang et al., 2014, 2015; Zhang, 2015; Wei et al., 2017; Shan et al., 2018) evaluated adverse events associated with CHM combined with WCM. The main symptoms reported were dry mouth, fatigue, sleep disorders, gastrointestinal complaints, dizziness, nausea, and flatulence. All of these SRs indicated that the side effects in CHM adjuvant therapy group were generally less than or lighter than that in WCM group.

#### Summary of Quality of Evidences

A total of 24 outcomes were measured by 6 included SRs (Kim et al., 2012; Wang et al., 2012; Zhang et al., 2014, 2015; Wei et al., 2017; Shan et al., 2018). Among these outcomes, the quality of evidence was high in 13 (54%), moderate in 10 (42%), low in 1 (4%), and very low in none (0%). Of the five downgrading factors, the risk of bias (*n* = 11, 46%) was the most common downgrading factor in the included SRs, followed by inconsistency (*n* = 3, 13%), imprecision (*n* = 0, 0%), publication bias (*n* = 0, 0%), and indirectness (*n* = 0, 0%). The details of quality of evidence in included SRs were generalized in Table 4.

**Table 4:** Summary of GRADE on evidences of primary outcomes.

**References**	**Certainty assessment**							**No. of patients**		**Effect**		**Certainty**	**Importance**
	**No.of studies**	**Study design**	**Risk of bias**	**Inconsistency**	**Indirectness**	**Imprecision**	**Other considerations**	**Trial**	**Control**	**Relative (95% CI)**	**Absolute (95% CI)**		
**UPDRS I SCORES**													
Wang et al. (2012)	5	R	Serious	Not serious	Not serious	Not serious	None	178	173	–	MD 0.33 lower (0.58 lower to 0.08 lower)	⊕⊕⊕○ Moderate	Important
Zhang et al. (2015)	9	R	Serious	Not serious	Not serious	Not serious	None	374	416	–	MD 0.68 higher (0.38 higher to 0.98 higher)	⊕⊕⊕○ Moderate	Important
Wei et al. (2017)	2	R	Not serious	Not serious	Not serious	Not serious	None	80	80	–	MD 0.40 lower (0.71 lower to 0.09 lower)	⊕⊕⊕⊕ High	Important
Shan et al. (2018)	4	R	Not serious	Not serious	Not serious	Not serious	None	218	182	–	MD 0.30 lower (0.54 lower to 0.06 lower)	⊕⊕⊕⊕ High	Important
**UPDRS II SCORES**													
Wang et al. (2012)	9	R	Serious	Not serious	Not serious	Not serious	None	395	391	–	MD 2.18 lower (3.03 lower to 1.33 lower)	⊕⊕⊕○ Moderate	Important
Zhang et al. (2015)	12	R	Serious	Not serious	Not serious	Not serious	None	524	566	–	MD 2.14 higher (1.66 higher to 2.62 higher)	⊕⊕⊕○ Moderate	Important
Wei et al. (2017)	5	R	Not serious	Not serious	Not serious	Not serious	None	168	164	–	MD 0.47 lower (0.69 lower to 0.25 lower)	⊕⊕⊕⊕ High	Important
Shan et al. (2018)	7	R	Not serious	Not serious	Not serious	Not serious	None	346	311	–	MD 2.21 lower (3.19 lower to 1.22 lower)	⊕⊕⊕⊕ High	Important
**UPDRS III SCORES**													
Wang et al. (2012)	12	R	Serious	Serious	Not serious	Not serious	None	522	511	–	MD 2.35 lower (4.61 lower to 0.08 lower)	⊕⊕○○ Low	Important
Zhang et al. (2015)	14	R	Serious	Not serious	Not serious	Not serious	None	639	682	–	MD 2.45 higher (2.03 higher to 2.86 higher)	⊕⊕⊕○ Moderate	Important
Wei et al. (2017)	5	R	Not serious	Serious	Not serious	Not serious	None	168	164	–	MD 0.35 lower (0.57 lower to 0.13 lower)	⊕⊕⊕⊕ High	Important
Shan et al. (2018)	8	R	Not serious	Serious	Not serious	Not serious	None	386	350	–	MD 3.26 lower (4.36 lower to 2.16 lower)	⊕⊕⊕⊕ High	Important
**UPDRS IV SCORES**													
Wang et al. (2012)	7	R	Serious	Not serious	Not serious	Not serious	None	238	236	–	MD 0.51 lower (0.83 lower to 0.20 lower)	⊕⊕⊕○ Moderate	Important
Zhang et al. (2015)	10	R	Serious	Not serious	Not serious	Not serious	None	362	406	–	MD 0.32 higher (0.15 higher to 0.49 higher)	⊕⊕⊕○ Moderate	Important
Wei et al. (2017)	3	R	Not serious	Not serious	Not serious	Not serious	None	94	97	–	MD 0.32 lower (0.60 lower to 0.03 lower)	⊕⊕⊕⊕ High	Important
Shan et al. (2018)	5	R	Not serious	Not serious	Not serious	Not serious	None	240	207	–	MD 0.18 lower (0.37 lower to 0.01 higher)	⊕⊕⊕⊕ High	Important
**THE TOTAL SCORE OF UPDRS**													
Wang et al. (2012)	10	R	Serious	Not serious	Not serious	Not serious	None	382	367	–	MD 60.9 lower (8.08 lower to 4.10 lower)	⊕⊕⊕○ Moderate	Important
Zhang et al. (2014)	4	R	Serious	Not serious	Not serious	Not serious	None	129	128	–	MD 7.35 lower (11.24 lower to 3.47 lower)	⊕⊕⊕○ Moderate	Important
Zhang et al. (2015)	10	R	Serious	Not serious	Not serious	Not serious	None	386	414	–	MD 6.18 higher (5.06 higher to 7.31 higher)	⊕⊕⊕○ Moderate	Important
Wei et al. (2017)	7	R	Not serious	Not serious	Not serious	Not serious	None	282	276	–	MD 0.35 lower (0.53 lower to 0.20 lower)	⊕⊕⊕⊕ High	Important
Shan et al. (2018)	10	R	Not serious	Not serious	Not serious	Not serious	None	269	234	–	MD 5.43 lower (8.01 lower to 2.86 lower)	⊕⊕⊕⊕ High	Important
**PDQ-39**													
Wei et al. (2017)	4	R	Not serious	Not serious	Not serious	Not serious	None	141	141	–	MD 0.35 lower (0.59 lower to 0.12 lower)	⊕⊕⊕⊕ High	Important
Shan et al. (2018)	3	R	Not serious	Not serious	Not serious	Not serious	None	91	92	–	MD 7.65 lower (11.46 lower to 3.83 lower)	⊕⊕⊕⊕ High	Important
**NMSQUEST**													
Shan et al. (2018)	2	R	Not serious	Not serious	Not serious	Not serious	None	74	73	–	MD 9.19 lower (13.11 lower to 5.28 lower)	⊕⊕⊕⊕ High	Important

*GRADE, Grade of Recommendation, Assessment, Development, and Evaluation; R: randomized trials; PDQ-39, Parkinson's Disease Questionnaire-39; NMSQuest, Non-Motor Symptoms Questionnaire; CHM, Chinese herbal medicine; CWM, Conventional western medicine*.

## Discussions

### Summary of Evidence

This overview indicated that a number of SRs of CHM for PD have emerged between 2006 and 2018, suggesting that the interest in the use of CHM for PD treatment has grown considerably in recent years. Compared with WCM, CHM paratherapy showed significant effect in improving UPDRS score, Webster scale score, PDQ-39, NMSQuest, CHM Syndrome Integral Scale, and PDSS. The side effect in CHM paratherapy group were generally less than or lighter than that in WCM group. The findings of present study supported the use of CHM paratherapy for PD patients but we should treat the evidence cautiously because of the methodological flaws. In addition, CHM monotherapy showed no difference relative to WCM according to various outcome measures.

### Limitations

SRs are considered as the highest level of evidence in healthcare; only data from SRs of high-quality RCTs will receive 1a-evidence according to the levels of evidence from the Center of Evidence-Based Medicine in Oxford (Glasziou et al., 2004). An overview of SRs is a comprehensive evaluation method, which summarizes the findings, detects the methodological quality, and grades the evidence quality of all included SRs on one disease. In this overview, a summary of the findings of included SRs showed that CHM paratherapy for PD has better efficacy and safety than that of WCM. However, there are some limitations in the present study. Firstly, most of the included SRs were based on the poor quality of primary studies. The reliability of positive results may be undermined by these methodological flaws. According to the AMSTAR, no prior design provided in all 11 studies which probably affected the rigor of SRs. Six studies failed to explain the interests conflicts, which may bring the difficulty to users to make the judgment on that whether the potential issues existed in SRs, such as anthropogenic factors caused by interests conflicts on evaluation outcomes. Secondly, the quality of evidence of most primary outcomes was moderate (42%) to high (54%). However, only 6 SRs provided full information for grading the quality of evidence, while the quality of evidence of remaining 5 included SRs were unclear, which may affect the comprehensiveness and convincingness of the result of quality grading. Thirdly, the included SRs mostly focus on the intermediate outcomes, such as UPDRS and Webster scale, which mainly reflect some point in the process of interventions affecting the disease, not fully reflect all results of complex pathological process, thus affecting the analysis of the effectiveness. Fourthly, PD is considered a multisystemic neurodegenerative disorder, together with motor symptoms and NMS. Recent researches indicate that some NMS are the direct results of PD progression, or induced by PD medication and increasing attention has been paid to NMS for PD patients (Antonini et al., 2015; Bastide et al., 2015; Shi et al., 2017). However, our included studies mainly focused on evaluating motor symptoms, ignoring the specific analysis of NMS (Schapira, 2015). Fifthly, various kinds of CHM existed in our included studies. Individual drugs have not been evaluated, so it was unclear what specific ingredient was effective.

### Implications

This is the first overview of SRs focused on the efficacy and safety of CHM for PD. In the 11 included SRs, CHM paratherapy exhibit significant improvement in PD symptoms compared with WCM. According to the safety assessment, the CHM for PD is generally safe and well-tolerated. The evidences available from the present study supported the use of CHM paratherapy for PD patients but we should treat that cautiously because of the methodological flaws. However, there is insufficient evidence of CHM monotherapy for PD.

Given the methodological issues, recommendations for further research are as follows: (1) when designing RCTs for CHM, some specific guidelines should be combined and used as a comprehensive guideline, such as the CONSORT 2010 statement (Schulz et al., 2010), guidelines for RCTs investigating CHM (Flower et al., 2012) and CONSORT for TCM (Bian et al., 2011); (2) in further RCTs for CHM, individual placebo-controlled group should be designed and studied to evaluate the placebo effect; (3) in order to evaluate the effectiveness of specific ingredient of CHM, further studies of the efficacy of individual CHM should be conducted; (4) it is important to improve the methodological quality of further SRs themselves. The PRISMA statement (Liberati et al., 2009) should be used as a guide and the prospective registration of SRs should be encouraged; (5) assessments of NMS are crucial and specific scales such as the Non-Motor Symptoms Scale, the Mini Mental State Examination, the Montreal Cognitive Assessment Test should be applied (Asakawa et al., 2016). The terminal outcomes in the natural course of PD can be more comprehensive, contributing to the more accurate evaluation of the efficacy of CHM for PD; (6) with the CHM being more widely used for PD, the reporting of adverse events may become more common, so we suggest that a special reporting format should follow up to ensure its safety.

## Conclusions

The findings of present study supported the use of CHM paratherapy for PD patients but we should treat the evidence cautiously because of the methodological flaws. Further rigor RCTs are still needed. In addition, there is insufficient evidence of CHM monotherapy for PD; however, it should be remembered that a lack of scientific evidence does not necessarily mean that the treatment is ineffective (Kotsirilos, 2005). Thus, study of CHM monotherapy for PD is open.

## Author Contributions

X-CJ, LZ, Y-YJ, and G-QZ designed the study. X-CJ, LZ, YW, H-BC, and X-JB contributed to the literature search, interpretation, writing, and proofreading of the manuscript. X-CJ, LZ, and YW extracted data and performed data analyses. X-CJ, LZ, and YW revised the study. H-BC and X-JB generated the figures.

### Conflict of Interest Statement

The authors declare that the research was conducted in the absence of any commercial or financial relationships that could be construed as a potential conflict of interest.
